# Ectopic pregnancy risk factors in infertile patients: a 10-year single center experience

**DOI:** 10.1038/s41598-022-24649-w

**Published:** 2022-11-28

**Authors:** Federico Cirillo, Ilaria Paladino, Camilla Ronchetti, Andrea Busnelli, Emanuela Morenghi, Leonora Grilli, Pasquale Patrizio, Elena Zannoni, Paolo Emanuele Levi-Setti

**Affiliations:** 1 Department of Gynecology, Division of Gynecology and Reproductive Medicine, Fertility Center, IRCCS Humanitas Research Hospital, Rozzano, Milan, Italy; 2 Department of Biomedical Sciences, Humanitas University, Pieve Emanuele, Milan, Italy; 3 Biostatistics Unit, IRCCS Humanitas Research Hospital, Rozzano, Milan, Italy; 4 Division Reproductive Endocrinology and Infertility, University of Miami, Miller School of Medicine, Miami, FL USA

**Keywords:** Reproductive disorders, Infertility

## Abstract

The present retrospective study included both intrauterine insemination and in vitro assisted reproductive technologies (ART) procedures performed from January 2009 to December 2018 at a tertiary-care Fertility Centre. The purpose was to assess the incidence of ectopic pregnancy (EP) in infertile population who undergoes ART and to identify any risk factor impacting the occurrence of EP after ART. Among 27,376 cycles, 7352 pregnancies were achieved, of which 132 were EPs, the 1.80% (95% CI 1.5–2.1) of all pregnancies. In fresh embryo transfer cycles, a history of prior pelvic adhesions showed the greatest impact on the incidence of EP (aOR 2.49 95% CI 1.53–4.07 p < 0.001). Other factors associated with EP incidence were also identified, such as female age, basal FSH, the transfer of blastocyst embryos and difficulties during the embryo transfer procedure. In frozen embryo transfer cycles, the only factor influencing the incidence of EP was anti Müllerian hormone (AMH) serum concentration (aOR 0.81 95% CI 0.65–1.00, p = 0.048). To conclude, the incidence of EP observed was comparable to that reported after natural conception. On the other hand, pre-existing risk factors, traditionally more common in infertile population, appeared to influence the incidence of EP and should thus be modified if possible.

## Introduction

Ectopic pregnancy (EP) is a leading cause of maternal death or morbidity during the first trimester of pregnancy^1,2^ EP stands not only for tubal location, even if it is the most common, but also for other less usual locations such as isthmic, cesarean scar, cervical, cornual, ovarian, and abdominal^3,4^. Even more rare case could be the occurrence of bilateral tubal EP^5^. While EP represents 1–2% of all pregnancies in the general population^1^, initial evidence demonstrated an increased incidence of EPs after assisted reproductive technologies (ART), reaching rates as high as 8.6%^6^. During the last years, significant improvements in ART techniques have been made and a decrease in the number of associated EPs has been observed. This is most likely due to a reduction in the number of transferred embryos^7^. Nonetheless, the incidence of EP still appears to exceed that observed after natural conception^8^. In addition, rates of EP are highly variable among centers of different countries (varying from 1.5 to 2% in UK and USA up to 5% in China)^7,9,10^ and in national registries^11–13^. It has thus been hypothesized that differences in ART procedures and protocols may play a role in justifying this incidence variability^14^. In particular, the stage of transferred embryos (cleavage versus blastocyst stage) and the impact of fresh versus frozen/thawed embryo transfer (ET) cycles^15–18^ have been widely investigated. In this regard, some studies found frozen ET to be associated with a slightly lower incidence of EP compared to fresh ET^19,20^. However, possible confounding factors cannot be ignored. In fact, EP and infertility notoriously share some risk factors, making it unclear whether ART procedures have a direct effect or if the observed association is due to the included population itself^21–23^. Furthermore, the specific role of uterine contraction during fresh or warmed cycles, uterine manipulation, progesterone for luteal phase support (LPS)^24,25^ and the operator performing the procedure still deserve to be investigated^26^. Considering the widespread use of ART worldwide and the high morbidity and mortality associated with EP, providing further insight into the risk factors associated with EPs after ART appears of utmost relevance. Against this background, the objective of the present study was to calculate the incidence of EPs in all medically assisted procreation (MAP)^27^ procedures (both intrauterine insemination and in vitro) at a single center throughout a 10-year period. In addition, we also attempted to identify risk factors impacting the occurrence of EP after MAP.

## Materials and methods

### Study design and population

In the present retrospective study, we included all MAP cycles (i.e., both intrauterine insemination and in vitro ART procedures) performed from January 1st, 2009 to December 31st, 2018 in a single, tertiary care, University-affiliated Infertility Unit (Fertility Center of the Humanitas Research Hospital, Rozzano, Milan, Italy). A total of 27,376 cycles in 11,686 couples were evaluated.

Collected data were female age, female body mass index (BMI), smoking habits, duration of infertility, primary or secondary infertility, previous history of EP, positive history for recurrent miscarriage, markers of ovarian reserve -i.e., basal follicle stimulating hormone (FSH), anti Müllerian hormone (AMH) and antral follicle count (AFC)-, positive history for pelvic adhesions (which refers to pelvic infections, endometriosis, pelvic adhesions and frozen pelvis), presence of uterine fibroids and cause of infertility. Additionally, for patients who underwent ART, we collected: the number of oocytes retrieved, the number of supernumerary frozen oocytes and embryos, the type of ET (i.e., fresh or frozen), the embryo stage at transfer (i.e., cleavage stage or blastocyst stage embryo), the number of fresh and frozen transferred embryos, and the difficulty of ETs. Since there is not a standardized definition of difficult transfer, ETs were considered difficult if they required more than one attempt, force, cervical manipulation or dilation, the use of a stylet or tenaculum or were associated with pain or bleeding^28^.

Considering the low number of EPs obtained during the study period, pelvic infections, endometriosis, pelvic adhesions and frozen pelvis were included in a single variable named positive history for pelvic adhesions.

Patients were divided in two sub-groups according to the MAP cycle outcome: positive outcome with EP and positive outcome with eutopic pregnancy according to the European Society of Human Reproduction and Embryology (ESHRE) terminology^29^.

### Interventions

#### Intrauterine insemination (IUI)

Ovarian stimulation protocol for IUI cycles and procedures have already been described elsewhere^30^. Patients were treated with human menopausal gonadotropin (HMG- Meropur, Ferring, Milan, Italy) or recombinant follicle stimulating hormone (rFSH-Puregon, MSD-Italy; Gonal-F, Merck Serono S.p.A., Rome, Italy) according to standard protocols and monitoring (75 IU/d starting from day 3 for a various duration depending on the ovarian response). An injection of urinary human chorionic gonadotropin (uHCG—Gonasi HP, Ibsa Italy) 5000 IU or recombinant human chorionic gonadotropin (rHCG—Ovitrelle; Merck Serono S.p.A.) 250 mg was given to trigger ovulation when the dominant follicle had average diameter of 18 mm or more. If three or more follicles of at least 15 mm were present, the cycle was canceled, and patients were instructed to refrain from unprotected sexual intercourse. On the day of IUI, a semen sample was obtained by masturbation after a 2–5 days of abstinence and collected in a sterile cup. Sperm capacitation was performed using density gradient centrifugation or swim-up procedure to remove seminal fluid and enhance sperm quality for IUI. A single IUI was performed 36 h post-HCG. The washed motile sperm population was concentrated in 0.5 mL and loaded in a soft catheter for insemination (Wallace Intrauterine Insemination catheter, Smiths Medical International, Australia) and the patient remained supine for 10 min after the procedure. Luteal phase was supported with 200 mg daily of micronized vaginal progesterone (Prometrium, Rottapharm S.pa. or Progeffik, Effik Italy S.p.a), starting on the same night of the IUI procedure.

#### In vitro fertilization (IVF): ovarian stimulation, egg collection and ET

Ovarian stimulation (OS) was performed using four different protocols: GnRH agonist long protocol; GnRH agonist short protocol; GnRH antagonist protocol; flare-up GnRH agonist protocol^31^. As previously described by the same research group^32^, most of antagonist OS started with the use of combined oral contraceptives pretreatment. Long GnRH agonist protocol was based on the administration of daily leuprorelin (Enantone die, Takeda, Italy) or Leuprolin acetate 20 IU/day (Fertipeptyl, Abbott Pharmaceutical Products, USA) on day 21 of the previous luteal phase of the stimulation cycle. When pituitary desensitization was achieved (14 days after the initiation of GnRH agonist), as evidenced by the absence of ovarian follicles > 5 mm and endometrial thickness < 5.4 mm on transvaginal ultrasound examination, gonadotropin stimulation was initiated. In short GnRH agonist protocol the agonist (Leuprolin 0.1 mg/day) was administered from day 21 of the previous cycled and induction from day one or two of the cycle (day one being the start of the menstrual bleed) reducing the agonist dose to 0.05 mg/day and continuing with stimulation until the day of HCG administration. In the GnRH antagonist protocol, the first day of women spontaneous menstrual cycle or a withdrawal bleeding after receiving a low dose oral contraceptive, gonadotropin stimulation was initiated and when the leading follicle reached 13–14 mm in mean diameter, and/or plasma E2 exceeded 400 pg/ml, an injection of 0.25 mg of GnRH antagonist (Cetrotide, Merck Serono S.p.A, Rome, Italy; Orgalutran, Organon, MSD-Italy) was administered by a subcutaneous injection (sc) daily until the day of ovulation trigger. Finally, in the flare-up GnRH protocol, daily agonist was provided on day 1 of the cycle with triptorelin (0.1 mg/day) and then gonadotropins were started according to ovarian reserve parameter on day 2 of the cycle. A starting variable dose of gonadotropin HMG (Meropur, Ferring, Milan, Italy) or rFSH (Puregon, MSD-Italy; Gonal-F, Merck Serono S.p.A., Rome, Italy) with or without the addition of recombinant luteinizing hormone (r-LH) for the first four days and then an individualized dose was administered according to the parameters resulting from transvaginal ultrasound and estradiol and progesterone levels until the day of ovulation trigger. The protocol of induction and the dose of gonadotropins administered were tailored on an individual basis according to the patient’s age, serum hormonal levels, and AFC. Transvaginal ultrasound, estradiol and progesterone determinations were performed from the 5th day of gonadotropin administration to monitor follicles and endometrial growth. When at least three follicles with a mean diameter of > 16 mm were observed and hormone levels were adequate, 250 μg of rHCG (Ovitrelle; Merck Serono S.p.A.) was administered subcutaneously, for final follicle maturation. Patients at high risk for ovarian hyperstimulation syndrome (OHSS) were triggered with a GnRH analogue (Decapeptyl, Ipsen, Milan, Italy or Fertipeptil, Ferring S.p.A, Milan, Italy)^33^. In these patients, if they did not receive 1500 IU of u-HCG at the time of trigger (dual trigger), the so called “Rescue Protocol” was performed for oocyte maturation by administering u-HCG 1500 IU (Gonasi HP, Ibsa Italy) on the day of oocyte retrieval together with either Micronized Progesterone (Prometrium, Rottapharm S.p.a., or Progeffik, Effik Italia S.p.a.) 200 mg × 2/day or Progesterone Gel (Crinone 8%, Merck, Serono) 80 mg × 2/day, and Estradiol Valerate (Progynova, Bayer, Schweiz, AG) 2 mg × 2/day. Oocyte retrieval was performed transvaginally 34–36 h after ovulation triggering. Fresh ET was performed either 3 or 5 days following oocyte retrieval according to the patient history and characteristics of the embryos. A standardized internal protocol for performing the ET was followed: freehand insertion of a preloaded soft catheter into the uterine cavity, under transabdominal ultrasound (US) guidance. The latter has always been performed throughout the study period, even prior to the NICE indication^34^. In cases of non-optimal endometrial growth, premature progesterone rise, increased risk of OHSS or pre-implantation testing, elective embryo cryopreservation was performed. Frozen-thawed ET was preceded by endometrial synchronization, using one of the following three protocols: natural cycle (NC-FET), modified natural cycle (mNC-FET) or artificial replacement cycle (AR-FET), as previously described by the same research group^35^. In brief, in natural cycles patients had serial transvaginal ultrasound monitoring starting between cycle day 8–12 to check for dominant follicle and endometrial development. LH testing started when a follicle with a mean diameter > 11 mm was identified. In patients with no positive urinary LH test despite a follicle of 16–20 mm and endometrial stripe of ≥ 7 mm, 5000 units of uHCG (Gonasi HP, Ibsa Italy) were administered (mNC). Embryo rewarming and transfer was planned 7 days after the spontaneous LH peak or HCG administration. Hormonal replacement cycles (AR-FET) consisted of oral estradiol valerate (E2V, 6–8 mg) (Progynova, Bayer, Schweiz, AG) from the second day of the menstrual cycle until the endometrial thickness reached at least 7 mm. No pre-treatment with GnRHa was used. The embryo transfer was scheduled after 5 days from the progesterone start with 600 mg of vaginal micronized progesterone tablets (Prometrium, Rottapharm S.p.a., or Progeffik, Effik Italia S.p.a., 200 mg every 8 h) or 180 mg of micronized progesterone vaginal gel (Crinone 8%, Merck, Serono, 90 mg twice a day). Exogenous progesterone supplementation was also started on the day of embryo transfer in the NC-FET group and 2 days after HCG administration in the mNC-FET group using 200 mg daily vaginal micronized progesterone tablets (Prometrium, Rottapharm S.p.a., or Progeffik, Effik Italia S.p.a.) or 90 mg micronized progesterone vaginal gel (Crinone 8%, Merck, Serono). Frozen blastocysts were rewarmed on the day of ET. On that day, patients were instructed to have a full bladder. ET was performed with patients in lithotomy position in a dark, pressure, humidity, and temperature-controlled surgical suite adjacent to the embryology lab. A dedicated flow cabinet with a warmed plate microscope was placed in the same room to reduce space and time between the embryologist and clinician. No anesthesia or other drug was used before or during any of the transfers. A sterile steel speculum was used to expose the cervix, to clean it with warm sterile physiological solution (NaCl 9% solution, Baxter, US) and to remove the vaginal and cervical mucus.

All the ET procedures were performed with transabdominal ultrasound guidance by a trained nurse.

### EP diagnosis

The group of EPs included different types of atypical locations: tubal, cervical, scar pregnancy, ovarian and more rarely abdominal sites. EP diagnosis was done by combining the clinical signs and symptoms, the beta human chorionic gonadotropins (ß-HCG) assessment and the transvaginal high-resolution ultrasound (TVU) imaging.

ß-HCG measurement was performed, according to the stage of transferred embryos, 12–14 days after fresh ET, Frozen Embryo Transfer (FET) or IUI procedures, and if positive was repeated every 48 h, until it reached at least 1000 IU.

TVU was performed at 6 weeks of gestation (4 weeks after the procedure) or earlier in case of patients presenting clinical signs and symptoms strongly suspicious for EP, such as abdominal pain, vaginal bleeding and abnormal rise of ß-HCG levels. Indeed an early detection and diagnosis of EPs is of great relevance for optimizing fertility preservation, especially in non-tubal EPs, thanks to minimally invasive treatment (e.g. hysteroscopy), alone or combined with medical treatment^36^.

### Primary and secondary aims

The primary aim of the present study was to detect the incidence of EP in patients who underwent intrauterine insemination and in vitro MAP procedures, after adjusting for potential multiple confounding factors. The secondary aim was to investigate the potential risk factors associated with EP occurrence after MAP procedures.

### Ethical issue

The study was conducted according to the Helsinki Declaration as well as in accordance with the current Italian legal and regulatory requirements. The study protocol design was conducted in accordance with Good Clinical Practice (GCP) guidelines of our internal Research Hospital, it was approved by the Humanitas Institutional Review Board (Comitato Etico dell’IRCCS dell’Istituto Clinico Humanitas) and the study protocol was registered in ClinicalTrials.gov (NCT04325854, 13/03/2020), prior to full variable extraction.

Patients who underwent these cycles signed an informed consent approving that their medical records could be used for research purposes, if their anonymity was protected. Hence no specific consent was needed for this study^23^. Patients provided an informed written consent to have data from their medical records used in research, as the internal IRB and ethics committee required.

Data used in this study were collected via an internal web-based database, which enables storage of information about any patient and provides easy access for data analysis. Data protection is warranted by advanced threat prevention, enterprise-class encryption, and authentication for any user with periodical need of password renewal^37^. All data were fully anonymized before the authors accessed them.

### Statistical analysis

First, EP trends in a 10-year period were evaluated by column charts and trend lines.

Then, a logistic regression analysis considering only pregnant patients (0 for eutopic pregnancy, 1 for ectopic pregnancy) was carried out. The analysis was conducted for all ART procedures (IUI and IVF) and subsequently restricted to the IVF population. In particular, the IVF population analysis was further restricted according to fresh and frozen embryo transferred cycles. We considered variables related to the infertile population risk factors and to the risk related to the procedure performed.

Results were expressed as mean ± standard deviation (SD), or median with interquartile range (IQR) or number and percentage, as appropriate. Association of ectopic pregnancy with all considered variables were explored through univariable logistic regression. Independent variables with a p value less than 0.25 were then submitted to a backward multivariable logistic regression analysis, to identify factors associated to outcomes. Some variables were not included in the multivariable analysis for lack of independence (eg. AMH and FSH) and only the most significative variables, or the ones with less missing data, were chosen.

Due to the strong impact of the "primary infertility" variable, and due to the not excessively high number of EPs, we decided to exclude this variable from the multivariable analysis, in order to better assess the impact of the others.

Results of the logistic regression analysis were expressed as odds ratio (OR), 95% confidence interval (CI)^31^.

All analyses were made with Stata15 (StataCorp, 2017. Stata Statistical Software: Release 15. College Station, TX: StataCorp LLC). Statistical significance was set at p < 0.05.

## Results

### Baseline characteristics and demographics

**Figure 1:**
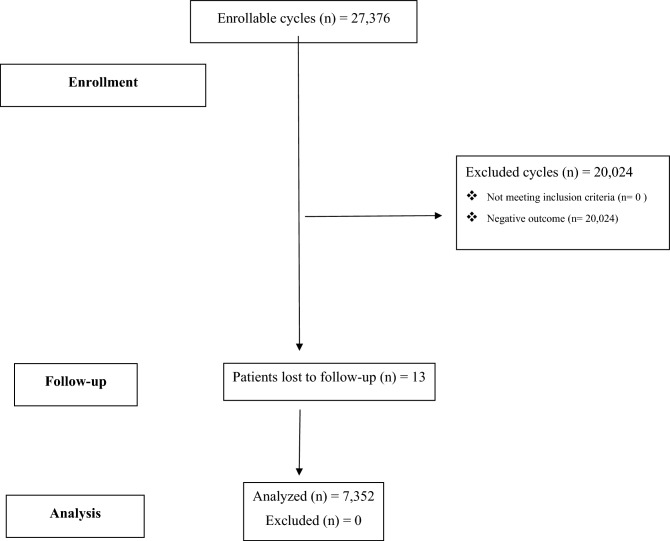
Study flowchart.

As shown in Fig. 1, a total of 27,376 cycles in 11,686 couples were evaluated. Of these, 16,119 were fresh ET cycles (58.9%), 6101 were FET cycles (22.3%), 1226 were oocyte warming cycles (4.5%) and 3930 were IUI cycles (14.3%). A total of 20,024 cycles resulted in a negative outcome (73.1%, 95% CI 72.6–73.7%), 132 resulted in EP (0.5%, 95% CI 0.4–0.6% of all cycles and 1.8%, 95% CI 1.5–2.1% of all pregnancies) and 7220 resulted in eutopic pregnancy (26.4%, 95% CI 25.9–26.9%). As for the sites of embryo implantation in the EPs, 113 (85.6%) were tubal, 6 cervical (4.6%), 4 ovarian (3%), 4 cornual (3%), 4 heterotopic (3%) and 1 not specified (0.8%). As for the treatment methods of EP, 78 (59%) were treated by salpingectomy, 24 (18.2%) by expectant management, 17 (12.9%) by methotrexate, 5 (3.8%) by dilation and curettage, 4 (3%) by ovariectomy, 2 (1.5%) by methotrexate and salpingotomy, 1 (0.8%) by dilation and curettage and salpingectomy, 1 (0.8%) by methotrexate and salpingectomy. Trends in EP rates over the years are reported in Supplementary Fig. 1 (EP% per two-year period for pregnant women). Patients’ demographic and clinical characteristics, of the whole study population and of the two subgroups separately, are reported in Table 1. Basal characteristics did not differ between groups with the exception of primary infertility incidence which was significantly higher in the eutopic pregnancy group, the basal FSH level which resulted significantly lower in the EP group and the rate of women with a positive history for pelvic adhesions which resulted significantly higher in the EP group. As for the indication to treatment, only endometriosis showed a significantly higher rate in EP group (Table 1). MAP outcomes in the general population and in the two sub-groups are reported in Table 2. No significant differences emerged with the exception of the embryo stage at transfer in fresh cycles (i.e., the cleavage stage ET was observed to be significantly more frequent in the eutopic pregnancy group) and the rate of difficult ETs which resulted significantly higher in the EP group.

**Table 1:** Population baseline characteristics.

Variable	All pregnancies	Ectopic pregnancy	Eutopic pregnancy	p value
Procedures	7352	132 (1.80%)	7220 (98.20%)	
Fresh ET	4506 (61.29%)	87 (65.91%)	4419 (61.20%)	
Frozen ET	2254 (30.66%)	32 (24.24%)	2222 (30.78%)	
Frozen oocytes	206 (2.80%)	3 (2.27%)	203 (2.81%)	
IUI	386 (5.25%)	10 (7.58%)	376 (5.21%)	
Female age (years)	35.65 ± 3.85	35.86 ± 3.77	35.65 ± 3.86	0.516
Female BMI (kg/m^2^)	21.45 (19.72–23.74)	21.48 (20.13–23.44)	21.45 (19.72–23.74)	0.798
Smoking (%)	2799 (38.07%)	56 (42.42%)	2743 (37.99%)	0.299
Infertility duration (years)	3.67 (2.50–5.42)	3.88 (2.58–5.58)	3.58 (2.50–5.42)	0.581
Primary infertility	3953 (53.77%)	46 (34.85%)	3907 (54.11%)	< 0.001
Previous EP	344 (4.68%)	4 (3.03%)	340 (4.71%)	0.365
Recurrent abortions	19 (0.26%)	0 (0.00%)	19 (0.26%)	0.555
Basal FSH (mUI/mL)	6.80 (5.50–8.30)	6.30 (5.25–7.60)	6.80 (5.50–6.80)	0.011
AMH	2.20 (1.20–3.87)	2.00 (1.12–3.31)	2.20 (1.20–3.90)	0.099
AFC	10 (6–17)	10 (6–15)	10 (6–17)	0.334
Positive history for pelvic adhesions^a^	961 (13.07%)	33 (25.00%)	928 (12.85%)	< 0.001
Uterine fibroids	1144 (15.46%)	28 (21.21%)	1116 (15.46%)	0.071
**Indication to treatment**				
Male factor	3044 (41.40%)	44 (33.33%)	3000 (41.55%)	0.057
Tubal factor	941 (12.80%)	22 (16.67%)	919 (12.73%)	0.180
Endometriosis	319 (4.34%)	11 (8.33%)	308 (4.27%)	0.031
Unexplained	950 (12.92%)	19 (14.99%)	931 (12.89%)	0.611
Male and female factor	987 (13.42%)	16 (12.12%)	971 (13.45%)	0.658
Ovulation disorder	225 (3.06%)	4 (3.03%)	221 (3.06%)	1.000
Diminished ovarian reserve	659 (8.96%)	11 (8.33%)	648 (8.98%)	1.000
Multiple female factors^b^	227 (3.07%)	5 (3.79%)	222 (3.07%)	0.606

BMI: body mass index; FSH: follicular stimulating hormone; AMH: anti Müllerian hormone; AFC: antral follicular count; ET: embryo transfer.

^a^i.e. pelvic infections, endometriosis, pelvic adhesions and frozen pelvis.

^b^More than one female factor, including genetic or recurrent pregnancy failures.

**Table 2:** IVF/ICSI fresh and frozen cycles outcomes.

Variable	All pregnancies	Ectopic pregnancy	Eutopic pregnancy	p value
Oocytes retrieved	9.69 ± 4.79	9.89 ± 4.65	9.69 ± 4.79	0.518
Cleavage stage transfers	4298 (61.88%)	78 (63.93%)	4220 (61.84%)	0.637
Blastocyst stage transfers	2648 (38.12%)	44 (36.07%)	2604 (38.16%)	
Cycles with frozen oocytes	532 (11.81%)	10 (11.49%)	522 (11.81%)	0.927
Cycles with frozen embryos	2066 (29.66%)	40 (32.79%)	2026 (29.60%)	0.445
Frozen embryos	1.89 ± 1.13	1.83 ± 1.17	1.89 ± 1.13	0.520
Frozen Oocytes	6.86 ± 2.91	7.90 ± 4.31	6.84 ± 2.88	0.960
Number Fresh Embryos Transferred	2.11 ± 0.59	2.06 ± 0.51	2.11 ± 0.59	0.347
Number Warmed Embryos Transferred	1.30 ± 0.55	1.49 ± 0.70	1.30 ± 0.55	0.089
Cleavage stage fresh embryo transfers	3925 (87.14%)	69 (79.31%)	3856 (87.30%)	0.027
Blastocyst stage fresh embryo transfers	579 (12.86%)	18 (20.69%)	561 (12.70%)	
Cleavage stage frozen embryo transfers	373 (15.27%)	9 (25.71%)	364 (15.12%)	0.084
Blastocyst stage frozen embryo transfers	2069 (84.73%)	26 (74.29%)	2407 (84.88%)	
Difficult transfers	1467 (21.47%)	35 (28.93%)	1432 (21.34%)	0.044

### Logistic regression analysis (ectopic pregnancy vs eutopic pregnancy)

First of all, we considered patients who underwent IUI and IVF (Table 3), and then we restricted our population exclusively to patients who underwent IVF, dividing them according to the type of ET cycle (i.e., fresh or frozen thawed) (Table 4). In supplementary Table 1 is shown the logistic regression (ectopic vs eutopic pregnancy) in only IUI cycles. Considering the entire population (i.e., patients who underwent IUI and IVF), both in univariable and multivariable logistic regression, factors which had a significant impact on EP incidence were the basal FSH concentration, (OR 0.91 95% CI 0.84–0.98, p = 0.012; aOR 0.90 95% CI 0.84–0.97, p = 0.010) and a positive history for pelvic adhesions, (OR 2.26 95% CI 1.52–3.37 p < 0.001; aOR 2.24 95% CI 1.50–3.37, p < 0.001).

**Table 3:** Univariable and Multivariable logistic regression (EP vs Eutopic pregnancy) in IUI, IVF/ICSI.

Variable	Univariable		Multivariable	
	OR (95% CI)	*p* value	OR (95% CI)	p value
Female age	1.01 (0.97–1.06)	0.543		
Female BMI	0.98 (0.93–1.03)	0.457		
Smoking	1.20 (0.85–1.70)	0.299		
Years of infertility	1.00 (0.98–102)	0.829		
Primary infertility	0.45 (0.32–0.65)	< 0.001		
Previous ectopic pregnancies	0.63 (0.23–1.72)	0.370		
Basal FSH	0.91 (0.84–0.98)	0.012	0.90 (0.84–0.97)	0.010
Basal AMH	0.93 (0.85–1.02)	0.124		
AFC	0.98 (0.95–1.01)	0.119		
Positive history for pelvic adhesions^a^	2.26 (1.52–3.37)	< 0.001	2.24 (1.50–3.37)	< 0.001
Uterine fibroids	1.47 (0.97–2.25)	0.072	−	
**Indication to treatment**				
Male factor	0.70 (0.49–1.01)	0.059	−	
Tubal factor	1.37 (0.86–2.18)	0.181		
Endometriosis	2.04 (1.09–3.82)	0.026		
Unexplained	1.14 (0.70–1.86)	0.611		
Male and female factor	0.89 (0.52–1.50)	0.658		
Ovulation disorder	0.99 (0.36–2.70)	0.984		
Diminished ovarian reserve	0.92 (0.49–1.72)	0.798		
Multiple female factors	1.35 (0.55–3.33)	0.518		
**Procedures**				
Fresh ET	1			
Frozen ET	0.73 (0.48–1.10)	0.133		
Frozen oocytes	0.75 (0.23–2.39)	0.628		
IUI	1.35 (0.70–2.62)	0.374		

A minus symbol in multivariable column indicates a variable entered in the analysis and then removed for lack of significance. Multivariable results are corrected by years.

IUI: intrauterine insemination; BMI: body mass index; FSH: follicular stimulating hormone; AMH: anti Müllerian hormone; AFC: antral follicular count; ET: embryo transfer.

^a^i.e. pelvic infections, endometriosis, pelvic adhesions and frozen pelvis.

**Table 4:** Univariable and Multivariable logistic regression (EP vs Eutopic pregnancy) in IVF/ICSI.

Variable	Fresh embryo transfer				Frozen embryo transfer			
	Univariable		Multivariable		Univariable		Multivariable	
	OR (95% CI)	p value	OR (95% CI)	p value	OR (95% CI)	p value	OR (95% CI)	p value
**Female age**	1.04 (0.99–1.11)	0.134	1.07 (1.01–1.13)	**0.033**	0.95 (0.87–1.03)	0.226		
**BMI**	0.98 (0.92–1.04)	0.477			0.96 (0.85–1.07)	0.421		
**Smoking**	1.06 (0.69–1.64)	0.783			1.31 (0.67–2.56)	0.433		
**Years of infertility**	1.00 (0.99–1.02)	0.785			0.95 (0.83–1.10)	0.515		
**Primary infertility**	0.42 (0.27–0.66)	**< 0.001**			0.61 (0.31–1.21)	0.157		
**Previous ectopic pregnancies**	0.54 (0.13–2.23)	0.398			0.89 (0.21–3.76)	0.878		
**Basal FSH (mUI/mL)**	0.88 (0.80–0.96)	**0.005**	0.87 (0.79–0.96)	**0.003**	0.96 (0.82–1.12)	0.598		
**Basal AMH**	1.01 (0.90–1.14)	0.820			0.77 (0.62–0.96)	**0.022**	0.81 (0.65–1.00)	**0.048**
**AFC**	0.98 (0.95–1.02)	0.336			0.97 (0.91–1.04)	0.413		
**Positive history for pelvic adhesions**^**a**^	2.35 (1.45–3.81)	**0.001**	2.49 (1.53–4.07)	** < 0.001**	2.21 (1.02–4.75)	**0.043**	−	
**Presence of myoma/s**	1.53 (0.91–2.55)	0.108	−		1.28 (0.55–2.94)	0.566		
**Indication to treatment**								
Male factor	0.71 (0.45–1.11)	0.128	−		0.79 (0.40–1.57)	0.503		
Tubal factor	1.44 (0.82–2.53)	0.206			1.55 (0.67–3.59)	0.301		
Unexplained	1.00 (0.50–2.00)	0.990			0.97 (0.34–2.78)	0.959		
Endometriosis	1.55 (0.67–3.61)	0.304			3.03 (1.05–8.76)	**0.040**	−	
Male and female factor	0.96 (0.52–1.77)	0.889			0.81 (0.28–2.30)	0.690		
Ovulation disorder	0.65 (0.09–4.71)	0.667			0.98 (0.13–7.22)	0.980		
Diminished ovarian reserve	1.09 (0.58–2.07)	0.781			NC			
Multiple female factors	1.57 (0.57–4.33)	0.388			0.98 (0.13–7.22)	0.980		
**Oocytes retrieved**	1.01 (0.97–1.05)	0.703						
**Embryos transferred**	0.85 (0.59–1.22)	0.376			1.64 (0.99–2.70)	0.051	−	
**Blastocyst stage transferred embryo**	1.34 (1.03–1.74)	**0.030**	1.32 (1.01–1.72)	**0.043**	0.72 (0.49–1.05)	0.089	−	
**Difficult transfers**	1.82 (1.16–2.85)	**0.009**	1.86 (1.18–2.93)	**0.007**	0.72 (0.28–1.87)	0.503		

A minus symbol in multivariable column indicates a variable entered in the analysis and then removed for lack of significance. Multivariable results are corrected by years.

BMI: body mass index; FSH: follicular stimulating hormone; AMH: anti Müllerian hormone; AFC: antral follicular count; ET: embryo transfer.

Significant values are in bold.

^a^i.e. pelvic infections, endometriosis, pelvic adhesions and frozen pelvis.

After limiting the analysis to patients who underwent IVF, we observed different results according to the type of ET (i.e., fresh ET vs frozen ET). In the fresh ET sub-group, factors found to have an impact on EP incidence both in univariable and multivariable logistic regression were basal FSH (OR 0.88 95% CI 0.80–0.96, p = 0.005; aOR 0.87 95% CI 0.79–0.96, p = 0.003), a positive history for pelvic adhesions (OR 2.35 95% CI 1.45–3.81, p = 0.001; aOR 2.49 95% CI 1.53–4.07 p < 0.001), blastocyst stage ET (OR 1.34 95% CI 1.03–1.74, p = 0.030; aOR 1.32 95% CI 1.01–1.72, p = 0.043) and a difficult transfer (OR 1.82 95% CI 1.16–2.85, p = 0.009; aOR 1.86 95% CI 1.18–2.93, p = 0.007). Female age resulted associated with an increased risk of EP only in the multivariable analysis (OR 1.04 95% CI 0.99–1.11, p = 0.134; aOR 1.07 95% CI 1.01–1.13, p = 0.033) (Table 4). On the other hand, the only factor which influenced EP incidence in the frozen ET sub-group was AMH (OR 0.77 95% CI 0.62–0.96, p = 0.022: aOR 0.81 95% CI 0.65–1.00, P = 0.048). Multivariable logistic regression analysis in Table 4 was adjusted per year.

## Discussion

The overall EP rate in our study conducted throughout a 10-year period, was 1.8% (95% CI 1.5–2.1) of all pregnancies. This rate is similar to the general population, where EPs account for 1–2% of all pregnancies^1^. Thus, our results do not suggest that patients who underwent MAP procedures have an increased risk of developing EP. The incidence noted in our study is also consistent with other recent contributions which evaluated EP rate in the ART population^7,9,38^.

Importantly, over the years, we observed a decrease in EP incidence after frozen ET (Fig. 1). This trend could be the consequence of the changes in the Italian law regulating ART (i.e., law 40/2004)^39^. In fact, in 2009, the lawmaker revoked both the insemination limit to a maximum of three oocytes and the obligation to transfer all the obtained embryos at the same time^39^. In the first following years, a progressive increase in the number of frozen-thawed ET cycles occurred. However, the policy of elective single blastocyst transfer took a few more years to take hold. One can thus speculate that the higher incidence of EPs observed in the first part of the study period is due to the widespread practice of transferring two or more frozen-thawed embryos. In fact, the analysis of data from the Centers for Disease Control and Prevention’s United States ART Surveillance System showed a progressive increase in the risk of EP as the number of transferred embryos increases^7^.

Having a positive history for pelvic adhesions was shown to have a relevant impact on the EP rate (aOR 2.24 95% CI 1.50–3.37, p < 0.001). The observed association was expected since tubal anatomy can be distorted after pelvic infections or endometriosis and it is well known that pelvic adhesions are a significant risk factor for EP in both natural and assisted conception^1,7,39^. In addition, the entity of this association was even more pronounced when the analysis was restricted to the fresh ET subgroup (aOR 2.49 95% CI 1.53–4.07 p < 0,001) (Table 4). This may be explained by the known modification of the hormonal balance. Indeed, the high estrogen levels may alter the uterine environment leading to a more pronounced uterine contractility and, as a consequence, to an increased risk of retrograde movement of the embryo in the fallopian tube^20,38,40–42^, even if no significant impact of the serum dosage of estradiol at the trigger day on EP was detected in fresh cycles (calculated per 100 pg/ml) (OR 1.01, 95% CI 1.00–1.03, p = 0.089; aOR 1.01, 95% CI 0.99–1.03, p = 0.228). Likewise, high progesterone levels are supposed to be responsible for a dysfunctional tubal peristalsis that may lead to a higher EP rate in fresh cycles^43^.

In our study, embryo transferred at blastocyst stage in fresh cycles emerged as a possible risk factor for EP (OR 1.34 95% CI 1.03–1.74, p = 0.030: aOR 1.32 95% CI 1.01–1.72, p = 0.043). A blastocyst has a higher implantation potential than a cleavage-stage embryo^44^. The combined effect of the higher blastocyst implantation potential and the increased uterine contractility in the fresh cycle environment may be a possible explanation to this finding. On the other hand, previous studies reported a reduced risk of EPs in frozen/thawed blastocyst ET^15,24,45^. Moreover, as reported in supplementary Table 2, notably if the number of transferred blastocysts was one, the p was not significant, so that it was possible to conclude that the transfer of blastocyst embryos in fresh cycles could significantly influence the risk of EP only if the number of transferred blastocysts is more than one. Single fresh blastocyst transfers did not increase the risk of ectopic pregnancy. The factors influencing the choice between cleavage or blastocyst stage embryo transfer greatly vary from one fertility clinic to another and may act as further confounding factors. Future studies considering possible covariates are thus warranted before drawing conclusion about the impact of embryo stage on the risk of EP.

Results about the association between biomarkers of ovarian reserve and EP risk are conflicting.

On one hand, we observed a lower EP risk in women in the high serum FSH category in fresh ET cycles (OR 0.88 95% CI 0.80–0.96, p = 0.005; aOR 0.87 95% CI 0.79–0.96, p = 0.003). This finding is not in line with most of the current literature^46–48^ showing either a higher risk of EP in women with high FSH serum concentration or the lack of an association^49–51^.

On the other hand, in the frozen ET group, high serum AMH concentration resulted associated with a reduction (OR 0.77 95% CI 0.62–0.96, p = 0.022; aOR 0.81 95% CI 0.65–1.00, p = 0.048). This finding agrees with the previous contribution on this issue^52^. Combining these contradictory findings into a unique explanation is a difficult task. Considering the non-negligible prevalence of this condition, more studies specifically designed to address this issue should be carried out.

A difficult or suboptimal ET (defined as an ET which required more than one attempt, cervical manipulation or that was associated with pain or bleeding) emerged as a possible risk factor in fresh cycles (aOR 1.86 95% CI 1.18–2.93, p = 0.007). It is well known that a difficult ET is associated with an increased risk of failed transfer. This occurs most commonly with a traumatic deposition of the embryo resulting from a difficult manipulation of the catheter inside the uterus, which may stimulate uterine contractions^53,54^. These uterine contractions have been linked to a relocation of the embryo once placed in the uterus^54,55^. The enhancement of uterine combinations favored by the concomitant ovarian stimulation could justify the observed association.

Therefore, a relevant attention should be put on the embryo transfer procedure, and on the possibility of better outcomes when the procedure is performed by an expert operator. This is described by a previous study which shows how the expertise of the operator performing embryo transfer is a crucial factor affecting the outcome of the ART cycle^26^.

Surprisingly, we failed to identify an association between tubal factor infertility and EP risk. Although tubal infertility is a known and well established risk factor for EP^1^, its evaluation may be difficult^56^. Tubal receptivity and abnormal tubal contractility cannot be assessed in every day clinical practice. Furthermore, according to our protocols, women who have an a priori indication to IVF don’t routinely undergo tubal patency evaluation. Henceforth, an underestimation of tubal factor infertility cannot be excluded making the observed association poorly reliable.

Our results did not show any statistically significant difference in the association between fresh or frozen ET and EP rates in both univariable and multivariable analysis. These are in line with previous research which also failed to show a relationship between EP and the type of ET^9,19^.

Despite the promising research efforts^57^, the EP early diagnosis is still made on the basis of the transvaginal sonography along with beta-human chorionic gonadotrophin monitoring, despite their well-known limits in accuracy, affordability, interpersonal variability and diagnostic readiness.

More specifically, reported sensitivity and specificity of TVUS for the detection of an ectopic pregnancy at a serum HCG of > 2000 milli-international units/mL are 10.9 and 95.2 percent, respectively^58^.

It is important to note that there is a variation in the level of HCG across pregnancies for each gestational age, and the discriminatory levels are not always reliable. In addition, other factors can affect the early detection of a gestational sac, including the skill of the ultrasonographer, the quality of the ultrasound equipment, and the presence of physical factors (eg, fibroids, multiple gestation, obesity).

### Strengths, limitations, need for future research and main conclusion of the study

The strengths of our study include the large number of cycles analyzed and the length of the study period. In addition, thanks to our strict protocols, patients lost at follow-up were few, 13 over 7,365 pregnancies (0.17%). Moreover, differently from other large studies which used national ART registries, our internal web-based database enabled us to collect information on established risk factors for EP, including previous obstetric and gynecological history, smoking habits, and uterine surgeries.

However, there are also some limitations. First, owing to the retrospective study design, several data in the dataset could be either conflicting or potentially incorrect. Previous ectopic pregnancies may be an instance of misleading data, due to missing information on the time of its occurrence.

In addition, we did not consider biochemical pregnancies in our study. Biochemical pregnancies are considered a very early pregnancy loss, i.e. a pregnancy that does not develop normally and that fails to progress^59^. Therefore, biochemical pregnancies may be regarded as an early EP which results in no implantation and miscarriage^60^. Lack of inclusion of biochemical pregnancies may have, thus, led to a possible EP incidence underestimation.

On the other hand, we might also have overestimated EP incidence due to our pregnancy follow-up protocol. In fact, we routinely perform ultrasonography four weeks after fresh ET, FET or IUI procedures (6th week of gestation). In contrast, in the general non-infertile population, first trimester obstetric ultrasound, in the absence of specific indications, is usually performed later. Several naturally conceived EPs which undergo spontaneous resolution are thus erroneously classified as spontaneous miscarriage or biochemical pregnancies. Furthermore, pregnancies conceived using ART may be monitored more closely, which may result in more frequent identification of EP than in spontaneously conceived pregnancies. It is also possible that some misclassification of ectopic pregnancies and miscarriages may have occurred, due to allocation of pregnancies of unknown location. In addition, in the present study it has not been analyzed the eventual impact of a conservative treatment (such as expectant management, medical management or fertility sparing surgical treatment) in case of intra- or extrauterine ectopic pregnancy on the obstetric outcomes, topic that surely needs further investigations.

## Conclusions

In conclusion, our study confirms that the EP prevalence of infertile women undergoing ART is comparable with that of naturally conceived pregnancies. On the other hand, pre-existing risk factors which are traditionally more common in the infertile population appear to influence the pregnancy outcomes. Pelvic infections, endometriosis, pelvic adhesions and frozen pelvis seem to play an important role in increasing the incidence of EP. In a clinical perspective, infertile patients should thus be evaluated for an EP risk factors assessment in order to actively reduce the modifiable risks factors. A special attention should be paid to women with a high a priori risk. In this subgroup, the embryo transfer procedure should be performed by an expert operator.

## Supplementary Information

Supplementary Figure 1.

Supplementary Tables.

## Data Availability

The dataset underlying this article is available in Zenodo and can be accessed only upon request because it includes sensitive data. Please address your request to the corresponding author if interested.

## References

[1] Marion LL, Meeks GR (2012). Ectopic pregnancy: History, incidence, epidemiology, and risk factors. Clin. Obstet. Gynecol..

[2] Khan KS, Wojdyla D, Say L, Gülmezoglu AM, Van Look PF (2006). WHO analysis of causes of maternal death: A systematic review. Lancet.

[3] Dialani V, Levine D (2004). Ectopic pregnancy: A review. Ultrasound Q..

[4] Hortu İ, A. L., Akdemir, A., Ergenoğlu, M., Yeniel, Ö. & Şendağ, F. Management of ectopic pregnancy in unusual location: Five-year experience in a single center. *J. Clin. Exp. Investig.* **8**(3), 90–95 (2017).

[5] Acet F, Goker ENT, Hortu I, Sahin G, Tavmergen E (2020). A rare case of bilateral tubal ectopic pregnancy following intracytoplasmic sperm injection-embryo transfer (ICSI-ET). Rev. Bras. Ginecol. Obstet..

[6] Clayton HB, Schieve LA, Peterson HB, Jamieson DJ, Reynolds MA, Wright VC (2006). Ectopic pregnancy risk with assisted reproductive technology procedures. Obstet. Gynecol..

[7] Perkins KM, Boulet SL, Kissin DM, Jamieson DJ, Group NASN (2015). Risk of ectopic pregnancy associated with assisted reproductive technology in the United States, 2001–2011. Obstet. Gynecol..

[8] Smith LP, Oskowitz SP, Dodge LE, Hacker MR (2013). Risk of ectopic pregnancy following day-5 embryo transfer compared with day-3 transfer. Reprod. Biomed. Online..

[9] Santos-Ribeiro S, Tournaye H, Polyzos NP (2016). Trends in ectopic pregnancy rates following assisted reproductive technologies in the UK: A 12-year nationwide analysis including 160 000 pregnancies. Hum. Reprod..

[10] Huang B, Hu D, Qian K, Ai J, Li Y, Jin L (2014). Is frozen embryo transfer cycle associated with a significantly lower incidence of ectopic pregnancy? An analysis of more than 30,000 cycles. Fertil. Steril..

[11] Centers for Disease Control and Prevention (CDC) (1995). Ectopic pregnancy–United States, 1990–1992. MMWR Morb. Mortal. Wkly. Rep..

[12] Society for Assisted Reproductive Technology - American Society for Reproductive Medicine (2007). Assisted reproductive technology in the United States: 2001 results generated from the American Society for Reproductive Medicine/Society for Assisted Reproductive Technology registry. Fertil. Steril..

[13] Scaravelli, G. *et al.* Attività del registro nazionale italiano della procreazione medicalmente. *Istituto Superiore di Sanità, Centro Nazionale di Epidemiologia, Sorveglianza e Promozione della Salute, Centro operativo adempimenti Legge 40/2004, Registro Nazionale della Procreazione Medicalmente Assistita.* http://www.salute.gov.it2019 (2017).

[14] Muller V, Makhmadalieva M, Kogan I, Fedorova I, Lesik E, Komarova E (2016). Ectopic pregnancy following in vitro fertilization: Meta-analysis and single-center experience during 6 years. Gynecol. Endocrinol..

[15] Fang C, Huang R, Wei LN, Jia L (2015). Frozen-thawed day 5 blastocyst transfer is associated with a lower risk of ectopic pregnancy than day 3 transfer and fresh transfer. Fertil. Steril..

[16] Li RR, Dong YZ, Guo YH, Sun YP, Su YC, Chen F (2013). Comparative study of pregnancy outcomes between day 3 embryo transfer and day 5 blastocyst transfer in patients with progesterone elevation. J. Int. Med. Res..

[17] Bu Z, Xiong Y, Wang K, Sun Y (2016). Risk factors for ectopic pregnancy in assisted reproductive technology: A 6-year, single-center study. Fertil. Steril..

[18] Shi W, Zhang W, Li N, Xue X, Liu C, Qu P (2019). Comparison of perinatal outcomes following blastocyst and cleavage-stage embryo transfer: Analysis of 10 years' data from a single centre. Reprod. Biomed. Online..

[19] Decleer W, Osmanagaoglu K, Meganck G, Devroey P (2014). Slightly lower incidence of ectopic pregnancies in frozen embryo transfer cycles versus fresh in vitro fertilization-embryo transfer cycles: A retrospective cohort study. Fertil. Steril..

[20] Londra L, Moreau C, Strobino D, Garcia J, Zacur H, Zhao Y (2015). Ectopic pregnancy after in vitro fertilization: Differences between fresh and frozen-thawed cycles. Fertil. Steril..

[21] Dubuisson JB, Aubriot FX, Mathieu L, Foulot H, Mandelbrot L, de Jolière JB (1991). Risk factors for ectopic pregnancy in 556 pregnancies after in vitro fertilization: Implications for preventive management. Fertil. Steril..

[22] Talbot P, Riveles K (2005). Smoking and reproduction: The oviduct as a target of cigarette smoke. Reprod. Biol. Endocrinol..

[23] Stewart LM, Stewart CJR, Spilsbury K, Cohen PA, Jordan S (2020). Association between pelvic inflammatory disease, infertility, ectopic pregnancy and the development of ovarian serous borderline tumor, mucinous borderline tumor and low-grade serous carcinoma. Gynecol. Oncol..

[24] Zeng MF, Li LM (2019). Frozen blastocyst transfer reduces incidence of ectopic pregnancy compared with fresh blastocyst transfer: A meta-analysis. Gynecol. Endocrinol..

[25] Xing W, Ou J, Cai L (2018). Thawed embryo transfer and ectopic pregnancy: A meta-analysis. Arch. Gynecol. Obstet..

[26] Cirillo F, Patrizio P, Baccini M, Morenghi E, Ronchetti C, Cafaro L (2020). The human factor: Does the operator performing the embryo transfer significantly impact the cycle outcome?. Hum. Reprod..

[27] Zegers-Hochschild F, Adamson GD, Dyer S, Racowsky C, de Mouzon J, Sokol R (2017). The international glossary on infertility and fertility care, 2017. Fertil. Steril..

[28] Levi Setti PE, Cirillo F, Morenghi E, Immediata V, Caccavari V, Baggiani A (2021). One step further: Randomised single-centre trial comparing the direct and afterload techniques of embryo transfer. Hum. Reprod..

[29] Kirk E, Ankum P, Jakab A, Le Clef N, Ludwin A, Small R (2020). Terminology for describing normally sited and ectopic pregnancies on ultrasound: ESHRE recommendations for good practice. Hum. Reprod. Open..

[30] Immediata V, Patrizio P, ParisenToldin MR, Morenghi E, Ronchetti C, Cirillo F (2020). Twenty-one year experience with intrauterine inseminations after controlled ovarian stimulation with gonadotropins: Maternal age is the only prognostic factor for success. J. Assist. Reprod. Genet..

[31] Bosch E, Broer S, Griesinger G, Grynberg M, Humaidan P, Ovarian Stimulation TEGG (2020). ESHRE guideline: Ovarian stimulation for IVF/ICSI. Hum. Reprod. Open..

[32] De Cesare R, Morenghi E, Cirillo F, Ronchetti C, Canevisio V, Persico P (2020). The role of hCG triggering progesterone levels: A real-world retrospective cohort study of more than 8000 IVF/ICSI cycles. Front. Endocrinol..

[33] Blumenfeld Z (2018). The ovarian hyperstimulation syndrome. Vitam. Horm..

[34] O'Flynn N (2014). Assessment and treatment for people with fertility problems: NICE guideline. Br. J. Gen. Pract..

[35] Levi Setti PE, Cirillo F, De Cesare R, Morenghi E, Canevisio V, Ronchetti C (2020). Seven years of vitrified blastocyst transfers: Comparison of 3 preparation protocols at a single ART center. Front. Endocrinol..

[36] Stabile G, Zinicola G, Romano F, Buonomo F, Mangino FP, Ricci G (2020). Management of non-tubal ectopic pregnancies: A single center experience. Diagnostics.

[37] Levi-Setti PE, Cirillo F, Scolaro V, Morenghi E, Heilbron F, Girardello D (2018). Appraisal of clinical complications after 23,827 oocyte retrievals in a large assisted reproductive technology program. Fertil. Steril..

[38] Jwa SC, Seto S, Takamura M, Kuwahara A, Kajihara T, Ishihara O (2020). Ovarian stimulation increases the risk of ectopic pregnancy for fresh embryo transfers: An analysis of 68,851 clinical pregnancies from the Japanese Assisted Reproductive Technology registry. Fertil. Steril..

[39] Chang HJ, Suh CS (2010). Ectopic pregnancy after assisted reproductive technology: What are the risk factors?. Curr. Opin. Obstet. Gynecol..

[40] Fanchin R, Ayoubi JM, Olivennes F, Righini C, de Ziegler D, Frydman R (2000). Hormonal influence on the uterine contractility during ovarian stimulation. Hum. Reprod..

[41] Mueller A, Siemer J, Schreiner S, Koesztner H, Hoffmann I, Binder H (2006). Role of estrogen and progesterone in the regulation of uterine peristalsis: Results from perfused non-pregnant swine uteri. Hum. Reprod..

[42] Zhang YL, Sun J, Su YC, Guo YH, Sun YP (2013). Ectopic pregnancy in frozen-thawed embryo transfer: A retrospective analysis of 4,034 cycles and related factors. Syst. Biol. Reprod. Med..

[43] Paltieli Y, Eibschitz I, Ziskind G, Ohel G, Silbermann M, Weichselbaum A (2000). High progesterone levels and ciliary dysfunction—A possible cause of ectopic pregnancy. J. Assist. Reprod. Genet..

[44] Glujovsky D, Blake D, Farquhar C, Bardach A (2012). Cleavage stage versus blastocyst stage embryo transfer in assisted reproductive technology. Cochrane Database Syst. Rev..

[45] Li Z, Sullivan EA, Chapman M, Farquhar C, Wang YA (2015). Risk of ectopic pregnancy lowest with transfer of single frozen blastocyst. Hum. Reprod..

[46] Patil M (2012). Ectopic pregnancy after infertility treatment. J. Hum. Reprod. Sci..

[47] Parashi S, Moukhah S, Ashrafi M (2014). Main risk factors for ectopic pregnancy: A case-control study in a sample of Iranian women. Int. J. Fertil. Steril..

[48] Kim SW, Kim YJ, Shin JH, Kim H, Ku SY, Suh CS (2019). Correlation between ovarian reserve and incidence of ectopic pregnancy after in vitro fertilization and embryo transfer. Yonsei Med. J..

[49] Malak M, Tawfeeq T, Holzer H, Tulandi T (2011). Risk factors for ectopic pregnancy after in vitro fertilization treatment. J. Obstet. Gynaecol. Can..

[50] Weigert M, Gruber D, Pernicka E, Bauer P, Feichtinger W (2009). Previous tubal ectopic pregnancy raises the incidence of repeated ectopic pregnancies in in vitro fertilization-embryo transfer patients. J. Assist. Reprod. Genet..

[51] Li C, Meng CX, Zhao WH, Lu HQ, Shi W, Zhang J (2014). Risk factors for ectopic pregnancy in women with planned pregnancy: A case-control study. Eur. J. Obstet. Gynecol. Reprod. Biol..

[52] Tan CW, Lee YH, Choolani M, Tan HH, Griffith L, Chan J (2013). Endometriosis, endometrium, implantation and fallopian tube. Hum. Reprod..

[53] Mains L, Van Voorhis BJ (2010). Optimizing the technique of embryo transfer. Fertil. Steril..

[54] Lesny P, Killick SR, Tetlow RL, Robinson J, Maguiness SD (1998). Embryo transfer–can we learn anything new from the observation of junctional zone contractions?. Hum. Reprod..

[55] Bulletti C, de Ziegler D (2006). Uterine contractility and embryo implantation. Curr. Opin. Obstet. Gynecol..

[56] Suresh YN (2014). The role of tubal patency tests and tubal surgery in the era of assisted reproductive techniques. Obstet. Gynaecol..

[57] Sahin C, Uygun ZO, Hortu I, Akdemir A, Kocamanoglu M, Ergenoglu AM (2021). Using dynein heavy chain 5 and creatine kinase levels in cervical fluid and blood for early diagnosing of ectopic pregnancy. J. Obstet. Gynaecol. Res..

[58] Condous G, Kirk E, Lu C, Van Huffel S, Gevaert O, De Moor B (2005). Diagnostic accuracy of varying discriminatory zones for the prediction of ectopic pregnancy in women with a pregnancy of unknown location. Ultrasound Obstet. Gynecol..

[59] Annan JJ, Gudi A, Bhide P, Shah A, Homburg R (2013). Biochemical pregnancy during assisted conception: A little bit pregnant. J. Clin. Med. Res..

[60] Zeadna A, Son WY, Moon JH, Dahan MH (2015). A comparison of biochemical pregnancy rates between women who underwent IVF and fertile controls who conceived spontaneously. Hum. Reprod..

