# Supplementary material for: Ectopic pregnancy risk factors in infertile patients: a 10-year single center experience
Source: Sci Rep. 2022 Nov 28;12:20473. doi: 10.1038/s41598-022-24649-w (PMC9705323; doi:10.1038/s41598-022-24649-w)
Supplement: Supplementary file 1 — Supplementary Figure 1. [file 41598_2022_24649_MOESM1_ESM.pdf]

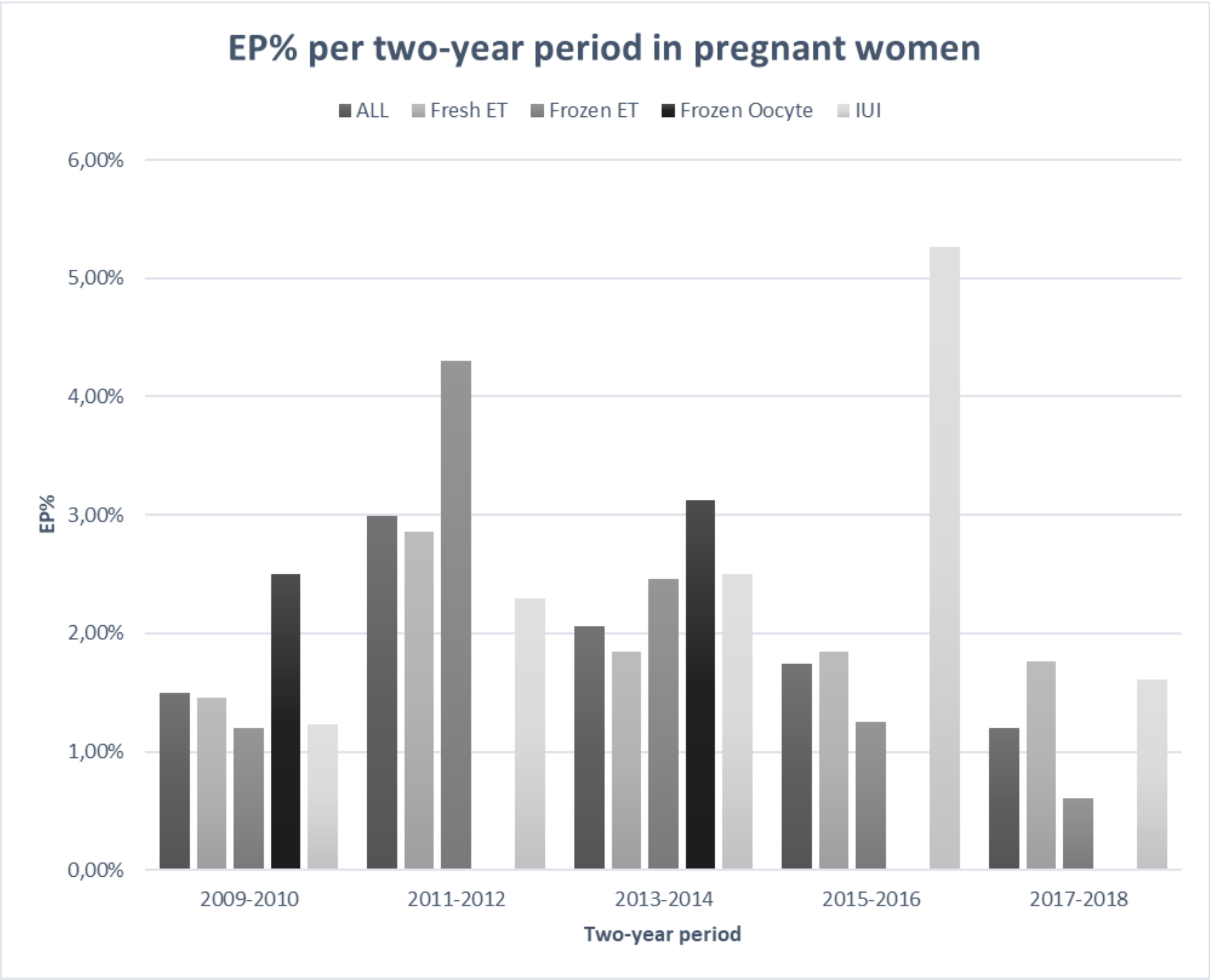

Supplementary Fig 1: EP% per two-year periods for pregnant women. EP: ectopic pregnancy; ET: Embryo Transfer; IUI: intrauterine insemination.
