# Supplementary material for: Ectopic pregnancy risk factors in infertile patients: a 10-year single center experience
Source: Sci Rep. 2022 Nov 28;12:20473. doi: 10.1038/s41598-022-24649-w (PMC9705323; doi:10.1038/s41598-022-24649-w)
Supplement: Supplementary file 2 — Supplementary Tables. [file 41598_2022_24649_MOESM2_ESM.pdf]

**Primary infertility**	0.23 (0.06-0.92)	0.038	0.17 (0.03-0.84)	0.029
**Previous Ectopic Pregnancies**	NC			
**Recurrent Pregnancy Failure**	NC			
**Basal FSH (mUI/mL)**	1.02 (0.74-1.42)	0.886		
**Basal AMH**	1.01 (0.82-1.23)	0.937		
**AFC**	0.98 (0.88-1.09)	0.742		
**Positive history for pelvic adhesions** [a]	10.33 (1.05-101.93)	0.046	31.14 (2.26-428.73)	0.010
**Presence of myoma/s**	2.44 (0.50-11.92)	0.272		
**Indication to treatment**				
Male factor	0.60 (0.07-4.80)	0.628		
Tubal factor	NC			
Endometriosis	5.11 (0.58-45.28)	0.143		
Unexplained	1.50 (0.42-5.40)	0.535		
Male and female factor	NC			
Ovulatory	1.06 (0.22-5.08)	0.946		
Reduced ovarian reserve	NC			
Multiple female factors	NC			
Total sperm count (10^6)	1.01 (1.00-1.01)	0.026	1.01 (1.00-1.01)	0.041
Progressive motility	0.97 (0.90-1.04)	0.395		
Semen normal form (%)	1.01 (0.80-1.27)	0.957		

Supplementary Table 1. Univariable and Multivariable logistic regression (ectopic vs eutopic pregnancy) in IUI. (BMI: Body Mass Index; FSH: Follicular Stimulating Hormone; AMH: Anti-Mullerian Hormone; AFC: Antral Follicular Count). Multivariable results are corrected by years.

[a] i.e. pelvic infections, endometriosis, pelvic adhesions and frozen pelvis.

1 transferred embryo	0.85 (0.44-1.66)	0.643
2 transferred embryos	1.61 (1.19-2.17	0.002

Supplementary Table 2. Pregnancy in transfer of blastocyst stage VS cleavage stage embryos per number of transferred embryos.
